# More Than Just a Bandage: Closing the Gap Between Injury and Appendage Regeneration

**DOI:** 10.3389/fphys.2019.00081

**Published:** 2019-02-08

**Authors:** Anneke D. Kakebeen, Andrea E. Wills

**Affiliations:** Department of Biochemistry, University of Washington School of Medicine, Seattle, WA, United States

**Keywords:** regeneration, *Xenopus*, limb bud, tail, reactive oxygen species, epigenetic, innate immune, proliferation

## Abstract

The remarkable regenerative capabilities of amphibians have captured the attention of biologists for centuries. The frogs *Xenopus laevis* and *Xenopus tropicalis* undergo temporally restricted regenerative healing of appendage amputations and spinal cord truncations, injuries that are both devastating and relatively common in human patients. Rapidly expanding technological innovations have led to a resurgence of interest in defining the factors that enable regenerative healing, and in coupling these factors to human therapeutic interventions. It is well-established that early embryonic signaling pathways are critical for growth and patterning of new tissue during regeneration. A growing body of research now indicates that early physiological injury responses are also required to initiate a regenerative program, and that these differ in regenerative and non-regenerative contexts. Here we review recent insights into the biophysical, biochemical, and epigenetic processes that underlie regenerative healing in amphibians, focusing particularly on tail and limb regeneration in *Xenopus*. We also discuss the more elusive potential mechanisms that link wounding to tissue growth and patterning.

## Introduction

Injuries that sever tissues such as the limb or spinal cord are met with radically different outcomes among vertebrates. In mammals, a limb amputation or spinal cord transection is followed by inflammation and fibrotic scarring that leaves the animal with a permanent disability. In urodele amphibians such as axolotls and newts, the same injury is followed by scarless regenerative healing that can fully restore both the lost tissue and its function (reviewed in Tanaka, 2016). The anuran frogs *Xenopus laevis* and *Xenopus tropicalis* represent a middle ground: injuries to the tadpole tail, limb bud, or spinal cord are readily repaired through regeneration, but this ability declines during metamorphosis (Cannata et al., 2001). As adults, *Xenopus* can no longer functionally recover from a spinal cord transection (Filoni and Bosco, 1981), while amputation of the hindlimb results in regeneration of a single digit, rather than the whole limb (DENT, 1962; Suzuki et al., 2006). This temporally restricted regenerative competence therefore makes *Xenopus* an appealing model for defining the features that enable or inhibit regenerative healing. In addition to the loss of regenerative competence undergone during metamorphosis, *Xenopus* tadpoles also experience a transient loss of regenerative competence called the refractory period at Nieuwkoop and Faber stages 45–47, shortly after the onset of independent feeding (Beck et al., 2003). Appendage regeneration, particularly of the tadpole tail, has been widely studied before, during and after this period. As a complement to the limb or limb bud, the tail is an excellent model for appendage regeneration because it comprises multiple cell types from epidermal, neural, mesodermal, and neural crest lineages, is easily accessible experimentally, and regenerates fully in a matter of days (Beck et al., 2009; Chen et al., 2014).

The regeneration of a tissue intuitively recapitulates aspects of its embryonic development. In both processes, rapid proliferation gives rise to new tissue, cell fate has to be specified within that tissue, and distinct positional identities have to be established to generate a properly patterned structure. Molecular evidence has validated multiple aspects of this parallel. Experimental perturbations using small molecule inhibitors and heat-shock inducible inhibitory proteins have established that BMP, FGF, Wnt, Notch, Shh, and Nodal/TGF-b signaling pathways are required for proper formation of the regenerated tail, paralleling their requirements in early embryonic patterning (Beck et al., 2003; Ho and Whitman, 2008; Lin and Slack, 2008; Taniguchi et al., 2014). Elegant experiments using heat-shock inducible expression of inhibitory proteins have further refined these observations to establish epistatic relationships, in which BMP acts upstream of Wnt, which in turn acts upstream of FGF during regeneration of the limb bud and tail (Lin and Slack, 2008). As during development, the establishment of positional identity appears to rely on the action of posterior Hox transcription factors (Christen et al., 2003). Numerous genes expressed in the developing limb and tail buds are re-expressed during tail regeneration, suggesting that many factors used to form these structures during embryogenesis are recapitulated during regeneration (Love et al., 2011; Chang et al., 2017).

**FIGURE 1:**
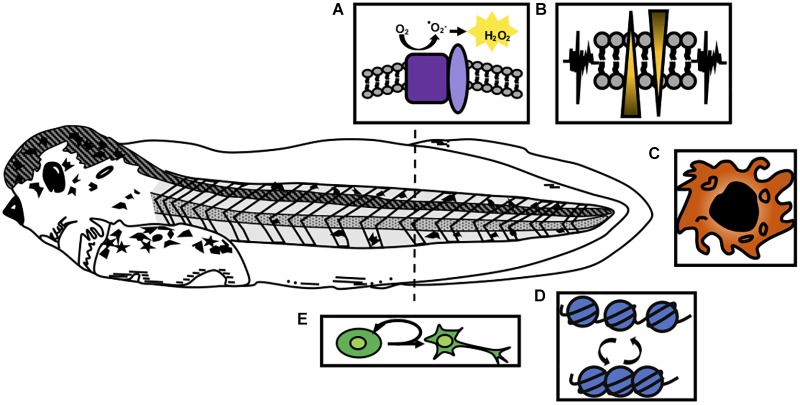
Cellular processes activated by injury in *Xenopus* tail regeneration. A regenerative stage 41 tadpole is shown, prior to the onset of independent feeding and the refractory period. Responses to injury that are critical for regeneration include **(A)** formation of reactive oxygen species such as H_2_O_2_ through the action of NOX complexes (purple) and p22-phox/cyba (light purple); **(B)** bioelectrical signaling mediated by ion channel activation; **(C)** recruitment of innate immune cell types such as macrophages; **(D)** epigenetic modifications that affect chromatin accessibility and transcription, and **(E)** activation of proliferation of blastemal cells and tissue-specific progenitors.

More recently, next-generation sequencing approaches have endeavored to comprehensively catalog the transcriptional responses undergone by regenerating tissues in *Xenopus*. Microarray and RNA-Seq studies of the whole regenerating tail (Love et al., 2011; Chang et al., 2017), proliferating blastemal cells (Tsujioka et al., 2015), and spinal cord (Lee-Liu et al., 2014), have highlighted that embryonic patterning and developmental processes are indeed highly prioritized beginning at 1 day after amputation. However, these studies show that the initial transcriptional responses triggered by injury include a distinct set of target genes that characterize regeneration independent from development, and also hold clues to how regenerative healing may be differentiated from other forms of wounding response. These include changes in cell metabolic enzymes, factors used to generate reactive oxygen species (ROS), ion channels, innate immune cell factors, and epigenetic modifiers (Figure 1). Functional interrogation of many of these cell physiological mechanisms has begun to confirm that they are not only upregulated but are also necessary for regeneration of the tail, limb, or spinal cord.

In this review, we examine emerging categories of intercellular and intracellular responses to complex tissue injury that are associated with the initialization of a regenerative program in *X. laevis* and *X. tropicalis*. We also explore emerging models for how the initial wounding responses might be coupled to activation of proliferation and patterning programs that allow these animals to fully restore lost structures.

## Rapid Changes in ROS Signaling and Membrane Potential Following Injury

**FIGURE 2:**
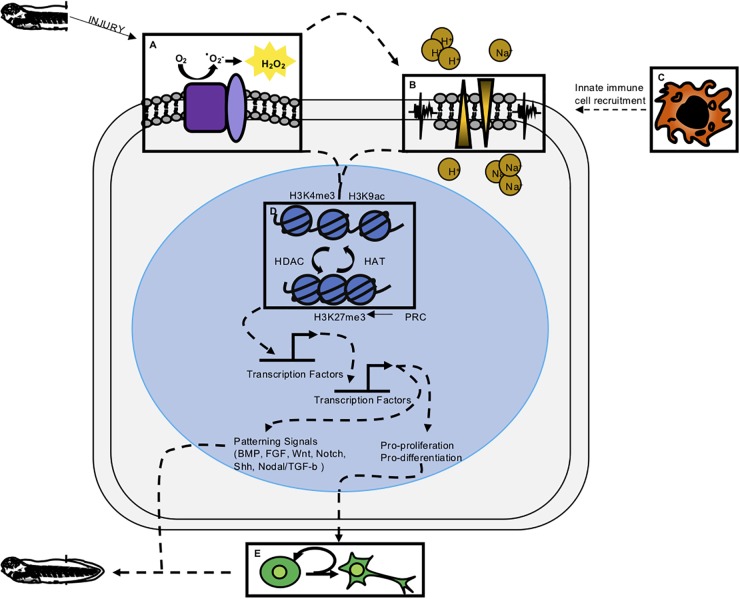
Integrative model of regeneration. Experimental evidence reviewed here suggests the five modalities of early wound response and regeneration discussed in this paper are interconnected. Experimentally determined, indirect connections are denoted by dashed lines. **(A)** ROS has been shown to be one of the most early activated signaling modalities and is upstream of ion channel activity, proliferation, epigenetic modification, and transcription factor activation. **(B)** Membrane depolarization and ion channel activity have been shown to act upstream of proliferation and innate immune cell recruitment. **(C)** Innate immune cells are shown to act upstream of proliferation and perhaps release cytokines necessary for successful regeneration. **(D)** Epigenetic modifications have been profiled to look at repressive and active marks in regeneration. Presence of these marks along with the epigenetic enzymes that remodel chromatin have been shown to be important for regeneration. **(E)** Cell proliferation appears to be downstream of early wound responses and perhaps part of a transition from wound repair to regeneration.

Following tail amputation, the first suite of signaling events reflect both short range intracellular damage responses and long-range signals activated by wounding. Among the earliest of these is ROS signaling, which can be detected using the H_2_O_2_-sensitive fluorescent reporter HyPER (Belousov et al., 2006; Figure 2A). In *X. laevis* that transgenically express HyPER, increased ROS is detectable within 20 min after tail amputation, and is strongly detectable by 6 h post amputation (hpa) (Love et al., 2013). Recently, it has been suggested that ROS production depends on a rapid influx of molecular oxygen from the surrounding environment, and that this influx is perturbed during the refractory period (Ferreira et al., 2018). Inhibition of ROS by treatment with the NADPH oxidase (NOX) inhibitors DPI or APO prevents full tail regeneration (Love et al., 2013; Ferreira et al., 2016, 2018) and inhibition of ROS more generally using free-radical scavengers such as MCI-186 delays regeneration. Morpholino knockdown of *cyba*, a member of NOX complexes 1, 2, and 4, also prevents regeneration, supporting the role of NOX complexes in this process (Love et al., 2013). Notably, DPI treatment prevents transcriptional activation of a Wnt reporter and of the Wnt target gene *fgf20*, suggesting that ROS is critical for activation of these later embryonic signaling pathways. HyPER activity is sustained for up to 4 days after injury, long after the closure of the wound epithelium. The prolonged activation of ROS, as well as transcriptional upregulation of ROS-associated pathway members (Love et al., 2011), suggest that ongoing production and response to ROS likely occur beyond the initial infiltration of atmospheric O_2_. It is not yet clear how ROS is sustained for long periods, or what cell types serve as the signaling source. Notably, while *spiB*-expressing innate immune cells such as macrophages are rapidly recruited to the injury site and are capable of producing ROS, *spiB* knockdown did not prevent ROS activation in the first few hours after injury (Love et al., 2013). However, innate immune cells may contribute to later phases of ROS signaling, as may the injured and newly regenerated tissues themselves.

Bioelectrical changes, particularly in membrane potential, are also rapidly triggered by amputation (Figure 2B). Shortly after tadpole tail amputation, staining with the membrane voltage dye DiBAC4(3) demonstrates depolarization of the regeneration bud (Adams et al., 2007). In the non-regenerative refractory period, this depolarization fails to occur. Depolarization is coincident with upregulation of the V-ATPase H+ pump in the regeneration bud, and when depolarization is prevented by inhibition of V-ATPase H+ pump function, regeneration fails to occur, implicating H+ ion flow as the source of membrane depolarization in this context. Activation of V-ATPase H+ pump activity improves regeneration during the refractory period (Adams et al., 2013), suggesting that this channel is both necessary and sufficient for induction of regeneration. V-ATPase H+ pump function is also required for appendage regeneration in zebrafish (Monteiro et al., 2014), suggesting a conserved role. The initial depolarization of the regeneration bud is transient, and by 24 hpa, an influx of sodium ions mediated by NaV1.2 is triggered following membrane repolarization (Tseng et al., 2010). Pharmacological inhibition of NaV1.2 activity also impedes regeneration, preventing proliferation, or activation of BMP and Notch pathways (Tseng et al., 2010). Other perturbations of membrane potential (V_mem_) similarly inhibit regeneration in tadpoles (Tseng and Levin, 2012) and in axolotls (Franklin et al., 2017). Numerous ion channels, including those for H^+^, Na^+^, K^+^, Ca^++^, and Cl^-^, are transcriptionally differentially expressed over the course of regeneration with variable temporal dynamics (Chang et al., 2017), suggesting that the role of ion channel activity in regeneration may be complex and interregulated with other mechanisms.

Bioelectrical signaling is also notable for its role in nerve conductance during regeneration. The nerve dependence of regeneration has long been noted in urodeles, dating from initial observations by Todd (1823). His descriptions of salamander hindlimb regeneration (or “re-production”) noted that excision or diversion of the sciatic nerve inhibited tissue growth, and also predicted that the nerve itself produced factors contributing to regenerative healing: “if the nerve be divided after reproduction has commenced, or considerably advanced, the new growth remains stationary, or it wastes, becomes shriveled and shapeless, or entirely disappears. This derangement cannot, in my opinion, be fairly attributed to the vascular derangement induced in the limb by the wound of the division, but must arise from something peculiar in the influence of the nerve (Todd, 1823).” Subsequent studies have confirmed that denervation of adult or larval limbs prevents cell proliferation and results in impaired regeneration (Singer and Craven, 1948), although this effect is abrogated in limbs where no nerve was initially present (Yntema, 1959), and can be rescued by addition of NRG1 (Farkas et al., 2016), or by activation of BMP and FGF signaling (Makanae et al., 2014). In zebrafish, nerve conductance is also critical to fin regeneration (Simões et al., 2014). In the late-stage *Xenopus* tadpole, peripheral nerves are required for limb bud regeneration (Cannata et al., 2001), while the spinal cord is required for tadpole tail regeneration (Taniguchi et al., 2008). Remarkably, there is evidence that bioelectrical signaling may also present a mechanism for long-range sensing and response to injury. After performing limb amputations, the uninjured contralateral limb also exhibits a rapid depolarization that mimics the timing and localization of the injured limb (Busse et al., 2018).

## Innate Immune Responses in Regeneration

Both regenerative and non-regenerative vertebrates respond to injury by recruiting innate immune cells to the injury site (Figure 2C). Transcriptomic studies of the regenerating tail and injured spinal cord implicate innate immune responses, which are highly upregulated beginning at 24 h post amputation (Lee-Liu et al., 2014; Chang et al., 2017). In *X. laevis*, transgenic fluorescent reporters in macrophage-like cells (*mpeg: mCherry*; *lurp: GFP* double positive) and neutrophil-like cells (*lurp: GFP* positive, *mpeg1: mCherry* negative) have been used to track cell behavior in these populations (Paredes et al., 2015). Fin injury is followed by rapid recruitment of both these cell types to the wound site, beginning with the migration of neutrophil-like cells to the injury site after 20 min, and followed by macrophage-like cells after an hour. This timing coincides closely with ROS activation. These cell infiltration behaviors were seen both in minor lateral fin injuries, such as a pin prick or hole punch, and in tail amputation.

Both neutrophils and macrophages act as producers of reactive oxygen signals and contribute to inflammatory responses, although neutrophils are associated with more inflammatory cytokine profiles (Kolaczkowska and Kubes, 2013). The role of neutrophils in regeneration has not yet been articulated clearly and may be variable (Rosales, 2018), but persistent infiltration of neutrophils at the wound site, as well as inflammatory signals such as nitric oxide (NO) that arise from these cells, is associated with scarring in mammals and may interfere with regenerative healing (Wilgus et al., 2013; Kryczka and Boncela, 2015). Pro-inflammatory agents such as beryllium sulfate also interfere with regeneration in *Xenopus* (Mescher et al., 2013). Macrophages serve as both a source and a responder cell type for ROS such as H_2_O_2_ and for numerous cytokines, several of which have now been explicitly investigated for their role in regeneration. Some of these, such as *mmp9* and *interleukin 7*, are transcriptionally activated both in regenerative and non-regenerative stages (Mescher et al., 2013). Certain macrophages are a source of pro-repair interleukins, such as IL6, which may contribute to regenerative healing. A transcriptomic study of proliferating blastemal cells in the regenerating tail identified the Il6 family member interleukin 11, and subsequent inducible gain-of-function experiments using CRISPR in *X. tropicalis* suggest that interleukin 11 acts upstream of stem cell proliferation in tail regeneration (Tsujioka et al., 2015, 2017).

Although the role of innate immune cells has not been deeply interrogated functionally in *Xenopus* regeneration, work from other species suggest that macrophages may be critical. In axolotls, peritoneal injection of the macrophage inhibitor clodronate results in impaired limb regeneration (Godwin et al., 2013). Macrophages are also critical for regenerative healing following cardiac injury in zebrafish (Lai et al., 2017), and for regenerative healing of dermal and cartilage injuries in the regenerative African spiny mouse *Acomys* (Simkin et al., 2017). A comparison of innate immune responses in *Acomys* and the non-regenerative house mouse showed that in both species, neutrophils are rapidly recruited to the wound site, but this is followed by infiltration from pro-repair M2 type macrophages only in *Acomys*, while in the house mouse, inflammatory M1 type macrophages are recruited, leading to greater tissue damage and scarring (Simkin et al., 2017). In *Xenopus*, it is not yet clear what molecular characteristics define populations or subpopulations of macrophages, or how these cell types contribute to regenerative versus non-regenerative healing. However, with the greater versatility of functional tools as well as genomic assessment tools now available in both *Xenopus* species, these are questions that can readily be addressed in the near future.

## Epigenetic Responses That Interpret Wounding Signals

A fundamental distinction in regenerative versus non-regenerative healing lies in the transcriptional response to injury (Figure 2D). Non-regenerative wound healing is followed by extensive fibrosis, characterized by dense cell matrix deposition and, in the spinal cord, by reactive gliosis. In regenerative healing fibrosis is minimized and proliferation and patterning of new tissue follows instead. How then, is the gene regulatory program associated with these latter behaviors activated? While transcriptomic analysis has shed considerable light on the plethora of genes that are both upregulated and downregulated during injury, experimental attention has also begun to turn to the epigenetic landscape that allows transcriptional activation of these targets. These experiments have included interrogating cell fate plasticity during regeneration, as well as investigations of chromatin marks and dynamics during regeneration.

In the axolotl, adult limb amputation and larval tail amputation are both followed by formation of a morphologically distinct blastema: a mass of highly proliferative mesenchymal cells with minimal morphological differentiation, protected by an apical epithelial cap that serves as a signaling source (McCusker et al., 2015). Because blastemal cells lack the clear morphological characteristics of differentiated cell types such as neurons or myocytes, it was long presumed that they represented a pluripotent cell type, analogous to embryonic stem cells. Indeed, in invertebrates that undergo morphallactic regeneration, such as planaria, blastemal cells have been clearly demonstrated to be pluripotent (Sánchez Alvarado, 2007; Aboobaker, 2011). However, lineage tracing experiments have made it clear that regenerating limb cells (in the axolotl) and tail cells (in *Xenopus*) retain a memory of their cell type of origin (Gargioli and Slack, 2004; Kragl et al., 2009). In both species, this was demonstrated by grafting specific tissues from a GFP-expressing donor into an unlabeled host, letting a full limb or tail develop, and then amputating the resulting, tissue-specifically labeled appendage. In axolotls, GFP-labeled muscle gave rise to muscle in the regenerated limb (but not skin or cartilage), labeled skin gave rise to skin (but not muscle or cartilage), and cartilage to cartilage (but not skin or muscle) (Kragl et al., 2009). It is worth noting that in axolotls, cartilage, and bone from the truncated limb do not contribute to the final regenerated skeleton, suggesting that other connective tissue cells are responsible for skeletogenesis, and therefore exhibit plasticity of fate (McCusker et al., 2016). Similar lineage tracing experiments in *Xenopus* examined animals where either the spinal cord, notochord, or muscle had been labeled with GFP, and found that only the same tissue was labeled following tail amputation and regeneration (Gargioli and Slack, 2004).

These grafting experiments suggested that cells might retain an epigenetically encoded memory of their tissue of origin. To begin to interrogate the nature of epigenetic memory, Hayashi et al. (2015a) collected limb bud tissue and analyzed the pattern of repressed and active promoter marks using ChIP-Seq for H3K27me3 and H3K4me3, respectively. They then profiled the epigenome of regenerated limb bud tissue 72 h after amputation, and found that genome-wide, most enhancers were similarly marked, including at genes associated with several signaling pathways critical for regeneration, including Shh and FGF. Notably, the Shh enhancer had previously been shown to gain methylation during metamorphosis, suggesting that methylation may contribute to loss of patterning gene expression during the loss of regenerative competence, although this hypothesis has not been queried more globally (Yakushiji et al., 2007). Hayashi and colleagues concluded that active enhancer marks were maintained at many enhancers throughout regeneration and might contribute to epigenetic memory. However, it should be noted that the temporal dynamics were not sampled at very high resolution in this study, and so it also remains possible that enhancer marks were instead re-established in the regenerating tissue. There may also be substantial variations in the epigenetic signature of specific cell types that have not yet been captured in studies like this one, which looked at the blastema in aggregate.

While lineage tracing experiments have implicated a stably maintained epigenetic memory in at least some regenerating tissues, other experiments have also demonstrated that epigenetic modifiers are required during regeneration, implicating dynamic regulation of chromatin marks and underlying transcriptional accessibility. DNA methylation and methyltransferases are dynamically regulated during limb regeneration in axolotls (Aguilar and Gardiner, 2015). In *Xenopus*, treating regenerating tails with the histone deacetylase (HDAC) inhibitors trichostatin A or valproic acid leads to minimal tissue regeneration and a loss of BMP target gene expression in the regenerate (Tseng et al., 2011; Taylor and Beck, 2012), and the HDAC inhibitor Sodium butyrate similarly inhibited regeneration (Tseng and Levin, 2012). These experiments suggest that HDACs are likely critical to tail regeneration. Acetylation serves to neutralize the positive change on lysine residues, which are abundant in histone tails and can form electrostatic interactions with negatively charged DNA. HDACs therefore generally have a chromatin closing effect by removing acetyl groups, while acetyl group deposition, carried out by histone acetyltransferases (HATs), generally acts to reduce chromatin density and increase accessibility. HAT activity is also affected during tail regeneration. To visualize dynamics of the activating mark H3K9ac, Suzuki et al. (2016) made use of an *in vivo* fluorescent mintbody, which demonstrated a ROS-dependent accumulation of H3K9ac in the notochord at 24 h of regeneration. Deposition or maintenance of the facultative heterochromatin mark H3K27me3 is also required: inhibition of the Polycomb Repressor Complex by treatment with DZNep results in a failure of limb bud regeneration (Hayashi et al., 2015b). The principal functional enzyme in the PRC is Ezh2, a methyltransferase that deposits the facultative heterochromatin mark H3K27me3 and serves to repress chromatin. These experiments demonstrate that enzymatic activities that serve both to increase and decrease chromatin accessibility are important for successful regeneration. They provide a first foray into defining epigenetic dynamics during regeneration, opening the door for ongoing functional and epigenomic analyses to define the timing, cell-type specificity, and genomic distribution of epigenetic modifications.

## Initiating Proliferation: Parallels to Tumorigenesis and the Role of Tissue-Specific Stem Cells

In *Xenopus*, amputation injuries are not immediately followed by upregulation of proliferation. Instead proliferation, marked by phospho-Histone H3 or by incorporation of nucleotide analogs, is increased strongly beginning at 1–2 days post amputation and continues through the remainder of tissue regeneration (Love et al., 2014; Tsujioka et al., 2015; Figure 2E). This timing is consistent with several relay steps being required between wounding and activation of proliferation, and is actually somewhat later than transcriptional activation of many patterning genes, including members of the Wnt, FGF, BMP, and Shh pathways (Love et al., 2011; Chang et al., 2017). Following tail amputation, proliferation occurs throughout the regenerating tailbud, and several studies have noted molecular similarities between the densely proliferating tail bud and tumorigenesis. Other tumorigenic signals are required for regeneration, including the Hippo pathway effector transcription factor YAP (Hayashi et al., 2014) and Shh (Taniguchi et al., 2014). Parallels to tumorigenesis have been more clearly articulated in the axolotl, where the physical resemblance of the loose mesenchymal blastema to a tumor has been described (Rojas-Muñoz et al., 2009), as have tumor activation programs (Knapp et al., 2013; Stewart et al., 2013). Activation of p53 also appears to be critical for proliferation in the axolotl, as pharmacological inhibition of p53 with nutlin or pifithrin impairs regeneration (Yun et al., 2013). The p53 amino acid sequence of axolotls has also been suggested to mimic that of activating mutations in human patients (Villiard et al., 2007).

There is reason to predict that there may be additional molecular parallels between tumorigenesis and blastema formation. In an intriguing hypothesis, Love et al. (2014) have noted that the pentose phosphate pathway, which serves to generate NADPH needed for NOX function, is also upregulated in cancer cells as part of the Warburg effect, where NADPH is required for biosynthesis of macromolecules needed for proliferation such as fatty acids and nucleotide precursors. This suggests an exciting potential metabolic link between ROS production and cell proliferation. In cancer cells, aerobic glycolysis is highly prioritized, and is enhanced by transcriptional upregulation of glycolytic enzymes induced by the hypoxia inducible factor HIF1a, which is itself stabilized in the hypoxic microenvironment of the tumor (Denko, 2008). Numerous growth factor inputs or oncogenes active in both cancer and regeneration, including PI3K/AKT, AMPK, p53, and Myc, stabilize or strengthen expression of the glycolytic and pentose phosphate pathway enzymes that perpetuate aerobic glycolysis and enable high rates of fatty acid and nucleotide biosynthesis (Vander Heiden et al., 2009). Although the metabolomics of regeneration are only beginning to be interrogated, similar transcriptional regulatory mechanisms may well be at play at least in *Xenopus* tail regeneration, where HIF1a is also critical (Ferreira et al., 2018) and ROS and NOX are activated.

In addition to widespread proliferation throughout the regenerate, there is clear evidence for tissue-specific stem cell proliferation. In *Xenopus* tail and spinal cord regeneration, Sox2/3-positive cells proliferate, contribute to new neurons and are required for regeneration of spinal cord form and function (Gaete et al., 2012; Muñoz et al., 2015). Birth dating of neurons by electroporation of Sox3:GFP into the tadpoles during regeneration provide evidence that Sox3 positive neural stem cells give rise to new neurons in regenerated tissue (Muñoz et al., 2015). Knockdown of Sox2/3 by morpholino or a dominant-negative construct impair regeneration of the spinal cord in stage 50 tadpoles (Gaete et al., 2012). The necessity for Sox2 specifically in spinal cord regeneration has also been found in axolotls by CRISPR mediated deletion of the gene, which results in impaired spinal cord regeneration but successful regeneration of other tissues (Fei et al., 2014).

While lineage tracing experiments have demonstrated that muscle cells retain cellular memory and become muscle cells in the regenerate, characterization of satellite cells has provided better resolution into the mechanism of muscle regeneration. Pax7 is a reliable marker of muscle satellite cells in *Xenopus*. Expression of *pax7* is upregulated in response to tail amputations. Experiments using a dominant-negative form of Pax7 (pax7EnR) did not significantly reduce muscle regeneration in response to an initial amputation, but secondary amputation of the regenerated tail that has been depleted of pax7 cells did show changes in muscle morphology, suggesting a role for satellite cells (Chen et al., 2006). Similarly, *pax7*-expressing cells have also been studied in axolotl muscle regeneration and have been shown to proliferate in response to damage and participate in regeneration (Morrison et al., 2006; Fei et al., 2017). The growing catalog of tools and markers to study specific cells will allow the field to increase the resolution into cell-type specific contributions to regeneration.

## Relationships Between Wounding Responses, and Looking Ahead to an Integrated Mechanism of Appendage Regeneration

It is now clear that immediate cellular injury responses are activated during regenerative healing, and that preventing them adversely affects regenerative outcomes. Researchers have begun to address how these responses are integrated with each other and with downstream effects on proliferation and patterning, and our present understanding of these relationships is summarized in Figure 2. Several studies have placed ROS activation as one of the most immediate responses to regeneration, upstream of changes in membrane potential, innate immune cell recruitment, proliferation, and patterning. In two separate studies, Ferreira and colleagues found that pharmacological inhibition of ROS after tail amputation prevented membrane depolarization and opening of Na^+^ channels (Ferreira et al., 2016, 2018), while activation of ROS during the refractory period was sufficient to restore V_mem_, channel activation and improved regenerative outcomes. This places ROS upstream of bioelectrical responses executed by ion channel activity (Figure 2A). In turn, inhibition of ion channel activity was shown to prevent macrophage recruitment to the injury site after tail amputation (Paré et al., 2017; Figure 2B,C). Similarly, ion channel activity is required for innate immune cell recruitment in axolotl regeneration (Franklin et al., 2017). Both ROS and ion-channel mediated depolarization act upstream of proliferation, which is not substantially upregulated until the second day after tail amputation. Proliferation is markedly decreased in tails treated with ROS-abrogating compounds (DPI, APO, MCI-186, Trolx) or channel inhibitors [DiBAC4(3)] (Adams et al., 2007; Tseng et al., 2010; Love et al., 2013; Ferreira et al., 2016, 2018).

At present, a major gap in our understanding of early regeneration lies in defining the transcription factors that interpret immediate wounding signals and couple them to epigenetic and transcriptional effects in the genome (Figure 2D). Several hypotheses have been advanced, summarized in Figure 2. These include the possibility that bioelectrical signals, possibly interpreted through regulated transport of sodium butyrate, may serve as an epigenetic modifier (Tseng and Levin, 2012; Pai et al., 2016), or that ROS may serve to activate transcription factor mobilization (Love et al., 2014). Support for the latter hypothesis recently came via the demonstration that the hypoxia-inducible factor HIF1A is turned on downstream of ROS during tail regeneration, and is required for activation of stress response genes such as hsp90 (Ferreira et al., 2018). HIF1A can also act as a transcriptional activator of numerous genes associated with processes important to regeneration, including angiogenesis, cell proliferation, and metabolic regulation, although the range of targets activated by HIF1A in regeneration is not yet known. In other regenerative species, ROS has been shown to act upstream of JNK and JAK/STAT (Santabárbara-Ruiz et al., 2015), and while these signaling pathways are required for tail or spinal cord regeneration in *Xenopus* (Sugiura et al., 2009; Tapia et al., 2017), a direct link between ROS and their activation has not yet been demonstrated in this context. Several lines of evidence therefore place epigenetic modification, and potentially several rounds of signaling, transcription factor binding activity, and transcriptional response, upstream of a relatively late burst in proliferation (Figure 2E). While these connections are promising, a more mechanistic dissection of how transcription factors are activated and their direct targets are needed in order to clarify how wounding is coupled to growth and patterning.

Our vision of regeneration has gained considerable nuance over the past decade, and is now beginning to fill in. Moving forward, focused attention is needed to define the mechanisms that link each required signaling modality to the next. This includes identifying how ROS and ion channel activities are sustained and regulated over regenerative time, and how their activation serves to recruit innate immune cells, as well as defining the subtypes of immune cells required for regeneration. We must also better define the cytokines or signals that directly mobilize transcription factors and epigenetic modifying enzymatic activities in the nucleus and define the direct targets of these factors. This latter step is critical to building an integrated picture of how upstream wounding events are coupled to the now vast amount of transcriptional data available in regeneration.

**FIGURE 3:**
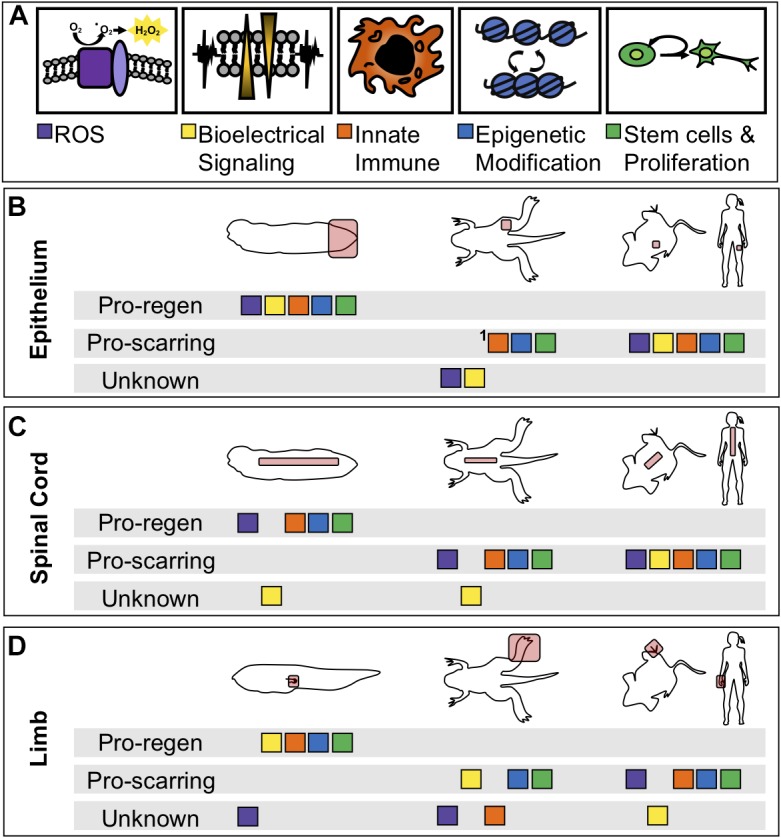
Comparison of physiological phenomena during healing between regenerative and non-regenerative organisms. **(A)** Color coded boxes correspond to either ROS (purple), changes in membrane potential (yellow), innate immune cell recruitment (orange), epigenetic reprogramming (blue), or tissue-specific stem cell proliferation and differentiation (green). **(B-D)** Summarization of physiological phenomena either known to be associated with regenerative healing, known to be associated with scarring, or not yet studied (unknown) in regenerative and non-regenerative contexts or species. “Pro-regen” refers to epimorphic regeneration as described in this review. “Pro-scarring” refers to injuries that undergo wound healing and scarring. **(B)** Regeneration or scarring of the epithelium in tadpoles (left), late-metamorphic froglets with minimal regeneration (middle), non-regenerative mammals (right). **(C)** Regeneration or scarring of the spinal cord. **(D)** Regeneration or scarring of the amphibian limb and mammalian digit tip. ^1^In adult frogs and mammals, the epidermal layer regenerates, however, the dermis is replaced by fibrous tissue.

More fundamentally, while many of these processes have been identified as critical in a particular tissue or at a particular stage, few have been systematically interrogated at multiple stages of tadpole development, during limb regeneration, in specific tissues such as spinal cord regeneration, and during regenerative loss in the refractory period and metamorphosis. The current state of sampling of each experiment is summarized in Table 1. As demonstrated in the table, early injury responses have been well studied in tail regeneration but are understudied in spinal cord or limb regeneration. The information we have learned about ROS, bioelectrical signaling, and immune cell response in the spinal cord have been suggested from expression profiles but have not been explicitly tested (Lee-Liu et al., 2014). Moreover, it is unclear what roles these modalities play in non-regenerative contexts (Figure 3). In the non-regenerative frog, very few of these modalities have been directly tested leaving many open questions. Are these modalities used by wound healing and scarring? Do they serve a different role than in regeneration? In the short term, we can use well studied mammalian wound healing as a guide for the use of these modalities in wound healing. In a non-regenerative mammalian context, an epithelial wound or digit tip wound are followed by re-epithelialization, inflammation, proliferation, and fibrotic scarring (Gurtner et al., 2008; Choi et al., 2017). A spinal cord injury slightly differs as it does not undergo re-epithelialization and the scar is a product of reactive gliosis (Ahuja et al., 2017). Each of these injury types have shown the participation of ROS, bioelectrical signaling, epigenetic modification and chromatin accessibility changes, and proliferation. Although we see parallels between mammals and non-regenerative frogs we cannot draw direct comparisons, making the evaluation of regenerative and non-regenerative stage *Xenopus* difficult. By linking our current models of regeneration at the wounding and patterning level, and by leveraging the unique utility of *Xenopus* for examining regeneration in multiple tissues and ages, we eagerly anticipate a clearer and therapeutically tractable understanding of how to activate regenerative healing.

**Table 1:** Summary of signaling modalities and known effects on regeneration.

Signal type	Observation	Method	Tail	Limb
ROS	ROS detected up to 4 dpi	HyPER	Belousov et al., 2006; Love et al., 2013	
	LOF	APO	Love et al., 2013	
	LOF, inhibition of fgf20 expression	DPI	Love et al., 2013; Ferreira et al., 2016, 2018	
	LOF	MCI186	Love et al., 2013; Ferreira et al., 2016	
	LOF	cybaMO	Love et al., 2013	
	Improved regeneration	Activation of ROS during refractory period	Ferreira et al., 2016, 2018	
	Perturbed influx of oxygen	Refractory period	Ferreira et al., 2018	
Bioelectrical	Depolarization of membrane detected	DiBAC4(3)	Adams et al., 2007	
	No depolarization	Refractory period	Adams et al., 2007	
	LOF	Inhibition of V-ATPase	Adams et al., 2013	
	GOF	Activation of V-ATPase in refractory period	Adams et al., 2007, 2013	
	Failure to proliferate, failure to activate BMP and Notch	Inhibition of NaV1.2	Tseng et al., 2010	
	Prevent macrophage recruitment	Inhibition of ion channel activity	Paré et al., 2017	
	LOF	Vmem perturbation	Tseng and Levin, 2012	
	LOF	Denervation	Singer and Craven, 1948	
	LOF	Peripheral nerve removal		Cannata et al., 2001
	LOF	Spinal cord removal	Taniguchi et al., 2008	
Innate immune	Impaired regeneration	Beryllium sulfate		Mescher et al., 2013
	LOF reduced cell proliferation GOF rescues LOF	IL-11 targeted CRISPR LOF IL-ll targeted CRISPR GOF	Tsujioka et al., 2017	
Epigenetic	Cells in blastema retain memory	Lineage tracing	Gargioli and Slack, 2004	
	Repressed and active promoter markers genome wide	ChIP-Seq for H3K27me3 and H3K4me3		Hayashi et al., 2015
HDAC	Impaired regeneration	TSA	Tseng et al., 2011; Taylor and Beck, 2012	Taylor and Beck, 2012
	Impaired regeneration, loss of BMP target expression	Valproic Acid	Tseng et al., 2011; Taylor and Beck, 2012	Taylor and Beck, 2012
	Impaired regeneration	Sodium butyrate	Tseng and Levin, 2012	
HAT	ROS-dependent accumulation of H3K9ac	*In vivo* fluorescent mintbody	Suzuki et al., 2016	
PRC	LOF	DZNep		Hayashi et al., 2015
Tumorigenesis/proliferation	Reduced proliferation, Impaired regeneration	Inhibition of p53: nutlin or pifithrin		Yun et al., 2013
	Impaired spinal cord regeneration, decrease in cell proliferation	Sox2MO/sox2 dominant-negative/sox2 CRISPR	Gaete et al., 2012; Muñoz et al., 2015	
	Rescues number of muscle satellite cells	pax7EnR	Chen et al., 2006	


## Author Contributions

AK contributed to the writing and editing of the manuscript, drafting and editing of the figures, and drafting and editing of the table. AW contributed to the writing and editing of the manuscript, and editing of the figures and table.

## Conflict of Interest Statement

The authors declare that the research was conducted in the absence of any commercial or financial relationships that could be construed as a potential conflict of interest.
